# Exploring the Psychological and Physiological Insights Through Digital Phenotyping by Analyzing the Discrepancies Between Subjective Insomnia Severity and Activity-Based Objective Sleep Measures: Observational Cohort Study

**DOI:** 10.2196/67478

**Published:** 2025-01-27

**Authors:** Ji Won Yeom, Hyungju Kim, Seung Pil Pack, Heon-Jeong Lee, Taesu Cheong, Chul-Hyun Cho

**Affiliations:** 1 Department of Psychiatry Korea University College of Medicine Seoul Republic of Korea; 2 School of Industrial Management Engineering Korea University Seoul Republic of Korea; 3 Department of Biotechnology and Bioinformatics Korea University Sejong Republic of Korea; 4 Department of Biomedical Informatics Korea University College of Medicine Seoul Republic of Korea

**Keywords:** insomnia, wearable devices, sleep quality, subjective assessment, digital phenotyping, psychological factors, mobile phone

## Abstract

**Background:**

Insomnia is a prevalent sleep disorder affecting millions worldwide, with significant impacts on daily functioning and quality of life. While traditionally assessed through subjective measures such as the Insomnia Severity Index (ISI), the advent of wearable technology has enabled continuous, objective sleep monitoring in natural environments. However, the relationship between subjective insomnia severity and objective sleep parameters remains unclear.

**Objective:**

This study aims to (1) explore the relationship between subjective insomnia severity, as measured by ISI scores, and activity-based objective sleep parameters obtained through wearable devices; (2) determine whether subjective perceptions of insomnia align with objective measures of sleep; and (3) identify key psychological and physiological factors contributing to the severity of subjective insomnia complaints.

**Methods:**

A total of 250 participants, including both individuals with and without insomnia aged 19-70 years, were recruited from March 2023 to November 2023. Participants were grouped based on ISI scores: no insomnia, mild, moderate, and severe insomnia. Data collection involved subjective assessments through self-reported questionnaires and objective measurements using wearable devices (Fitbit Inspire 3) that monitored sleep parameters, physical activity, and heart rate. The participants also used a smartphone app for ecological momentary assessment, recording daily alcohol consumption, caffeine intake, exercise, and stress. Statistical analyses were used to compare groups on subjective and objective measures.

**Results:**

Results indicated no significant differences in general sleep structure (eg, total sleep time, rapid eye movement sleep time, and light sleep time) among the insomnia groups (mild, moderate, and severe) as classified by ISI scores (all *P*>.05). Interestingly, the no insomnia group had longer total awake times and lower sleep quality compared with the insomnia groups. Among the insomnia groups, no significant differences were observed regarding sleep structure (all *P*>.05), suggesting similar sleep patterns regardless of subjective insomnia severity. There were significant differences among the insomnia groups in stress levels, dysfunctional beliefs about sleep, and symptoms of restless leg syndrome (all *P*≤.001), with higher severity associated with higher scores in these factors. Contrary to expectations, no significant differences were observed in caffeine intake (*P*=.42) and alcohol consumption (*P*=.07) between the groups.

**Conclusions:**

The findings demonstrate a discrepancy between subjective perceptions of insomnia severity and activity-based objective sleep parameters, suggesting that factors beyond sleep duration and quality may contribute to subjective sleep complaints. Psychological factors, such as stress, dysfunctional sleep beliefs, and symptoms of restless legs syndrome, appear to play significant roles in the perception of insomnia severity. These results highlight the importance of considering both subjective and objective assessments in the evaluation and treatment of insomnia and suggest potential avenues for personalized treatment strategies that address both psychological and physiological aspects of sleep disturbances.

**Trial Registration:**

Clinical Research Information Service KCT0009175; https://cris.nih.go.kr/cris/search/detailSearch.do?seq=26133

## Introduction

Insomnia is a common health complaint of all age groups, affecting millions of people worldwide. At the clinically pathologic end of the insomnia spectrum lies the insomnia disorder. Insomnia disorder is defined as dissatisfaction with sleep quantity or quality that causes clinically significant distress, with a minimum of 3 nights per week for at least 3 months with adequate sleep opportunity, by the *Diagnostic and Statistical Manual of Mental Disorders, Fifth Edition* [1]. *The International Classification of Sleep Disorders, Third Edition*, another widely used diagnostic tool for insomnia disorder, defines chronic insomnia disorder as nighttime sleep difficulty with related daytime problems that occur at least 3 times per week for at least 3 months with adequate opportunity and circumstances for sleep [2]. In the setting of primary care medicine, about 40% of patients reported significant sleep disturbances [3]. A total of 30% of adults reported any insomnia symptoms, and 10% met the criteria for insomnia disorder with daytime impairment included [4,5]. According to a nationwide population-based retrospective cohort study in South Korea, the prevalence of sleep disorders increased from 7.62% in 2011 to 14.41% in 2020, and insomnia was the most predominant sleep disorder diagnosis with a proportion of 91.44% [6].

The Insomnia Severity Index (ISI) is a widely used self-reported questionnaire that assesses the severity of insomnia symptoms and their impact on everyday life [7]. It is a useful questionnaire for research and in the clinical setting, and it has acceptable validity and reliability for evaluating and screening for primary insomnia [8]. Insomnia is a subjective health complaint that can be affected by a number of factors, including a stress event, recent transmeridian travel, medical condition, mood state, substance use, and work schedule. Of the possible factors, a cognitive phenomenon called “sleep state misperception” might affect one’s perception of sleep as well [9]. Sleep state misperception, which is also known as “paradoxical insomnia or subjective insomnia,” is a condition where one perceives his or her sleep to be worse than it actually is. There is often a discrepancy between subjective and objective sleep assessments; for example, underestimation of total sleep time and overestimation of sleep onset latency and wake after sleep onset are the usual presentations [10]. We first wanted to look into the nature of the subjectivity the ISI captures by evaluating the objective sleep-related parameters collected by wearable devices.

While previous studies have largely focused on clinical evaluations and polysomnography-based assessments, our research distinguishes itself by using wearable technology to collect data in a natural environment over an extended period [11,12]. This approach allows us to gain insights into how subjective sleep complaints may differ from objective measures, providing a more comprehensive understanding of insomnia’s multidimensional nature. Wearable technology has recently emerged as a valuable tool for monitoring sleep patterns in real-world environments. Using wearable devices in this study allows us to collect continuous and objective sleep data, minimizing the impact of the artificial setting of a sleep laboratory and providing a more realistic understanding of sleep behaviors [13]. Wearable devices offer the capability to track sleep duration, sleep stages, and circadian rhythm patterns over extended periods [14-16], making them particularly useful for identifying discrepancies between subjective sleep perceptions and actual sleep behaviors.

Fitbit, one of the widely used wearable devices, was first introduced in the early 2000s for health-conscious consumers. Early-generation Fitbit models provided sleep parameters, and recent-generation models now estimate sleep parameters, stage, and time based on the refined algorithm on body movement and heart rate variability. There have been concerns and studies for the validity of Fitbit against the gold standard polysomnography in measuring sleep parameters [14,17,18]. According to a recent review study, the recent-generation Fitbit models showed no significant difference in measured values of wake after sleep onset, total sleep time, and sleep efficiency, and they showed a sensitivity of 0.95-0.96 and specificity of 0.58-0.69 in detecting sleep epochs [19]. Fitbit Inspire 2, one of the recent-generation Fitbit models, significantly overestimated total sleep time, deep sleep time, and rapid eye movement [REM] sleep time; overestimated time in bed, sleep efficiency, and wake after sleep onset; and underestimated light sleep [20]. It showed a high sensitivity of 0.94 and a low specificity of 0.13, with an accuracy of 0.76 when compared with the polysomnography [20]. Considering the limitations of Fitbit, it cannot replace polysomnography; however, it is a useful, cost-efficient, and easily accessible tool for clinical research.

We then aimed to assess multidimensional variables across the no insomnia group and insomnia groups of all severities determined by the ISI scores to identify factors that affect subjective insomnia and its severity. The purpose of this study is to explore the relationship between subjective insomnia severity, as assessed by the ISI, and objective sleep parameters obtained through wearable devices. Specifically, we aim to determine if discrepancies exist between subjective reports of insomnia and activity-based objective sleep quality and to identify key factors that influence these discrepancies.

## Methods

### Study Participants

A total of 250 study participants were recruited from Korea University and Datamaker from March 2023 to November 2023 through advertisements on internet communities and campus noticeboards. The participants included both individuals with and without insomnia aged 19-70 years with the following inclusion criteria: more than 3 days a week of subjective insomnia symptoms during the past 3 months and an ISI score greater than 15 for the insomnia group, and fewer than 3 days a week of subjective insomnia symptoms during the past 3 months and an ISI score less than 8 for the no insomnia group. The exclusion criteria for the insomnia group were the following: history of intellectual disability or sign of organic brain injury; diagnosis of schizophrenia spectrum disorder; currently treated for a sleep disorder, such as narcolepsy, sleep-related breathing disorder, and parasomnia; and a smartphone nonuser. In addition to these, the exclusion criteria for the no insomnia group included the diagnosis of major mood disorder and anxiety disorder, shift worker, and current user of anxiolytic-sedatives or sleep-related functional foods.

The study participants provided informed consent before the study enrollment and completed the baseline assessments including demographic information; current and past medical and psychiatric history for all participants; and current medications, specifically sleep-related medication and functional foods for the insomnia group. In addition, the study participants were provided with wearable devices (Fitbit Inspire 3, Fitbit Inc) for the collection of step counts, heart rate, and sleep information. The participants also used a smartphone app named SOMDAY (Lumanlab Inc) as an ecological momentary assessment tool. They downloaded the smartphone app and recorded information on their daily alcohol consumption, smoking, caffeine intake, food consumption, and exercise.

### Assessments

#### Subjective Data: Self-Reported Questionnaires and Ecological Momentary Assessment Using SOMDAY

##### Self-Reported Questionnaires

In addition to the baseline assessment mentioned in the *Study Participants* section, the following self-reported questionnaires were completed by the study participants: the ISI [7], International Restless Legs Scale (IRLS) [21], Dysfunctional Beliefs and Attitudes About Sleep (DBAS) [22], State-Trait Anxiety Inventory–State (STAT-S) [23], the Korean version of Patient Health Questionnaire-9 (PHQ-9) [24], the Korean version of Generalized Anxiety Disorder-7 (GAD-7) [25], the Korean version of Alcohol Use Disorders Identification Test-Consumption (AUDIT-C) [26], Smartphone Overuse Screening Questionnaire (SOS-Q) [27], and the Korean version of the Biological Rhythms Interview of Assessment in Neuropsychiatry (BRIAN) [28].

##### Insomnia Severity Index

The ISI is composed of 7 items rated on a 5-point Likert scale with a total score ranging from 0 to 28. The 7 items include the following: the severity of difficulty falling asleep, difficulty maintaining sleep, early morning awakening, the satisfaction level of sleep, the severity of daytime activity impairment, the severity of disturbed quality of life observed by others, and the severity of worry about sleep problems. The score is interpreted as no clinically significant insomnia (scores 0-7), mild insomnia (scores 8-14), moderate clinical insomnia (scores 15-21), and severe clinical insomnia (scores 22-28).

In this study, the ISI was conducted twice on each study participant for different purposes. The first assessment of ISI was conducted at the screening stage in order to examine the inclusion and exclusion criteria. We decided to use the ISI scores higher than 15 for the insomnia group to secure study participants with subjective insomnia with at least moderate severity. Likewise, we defined the ISI score as less than 8 for the no insomnia group to ensure that the study participants in this group had no clinically significant subjective insomnia. The second assessment of ISI was performed after enrollment in order to measure the severity of subjective insomnia at the point of assessment.

##### International Restless Legs Syndrome Rating Scale

The IRLS is a 10-item self-reported questionnaire assessing the frequency, severity, and effect of restless legs syndrome (RLS). The total score ranges from 0 to 40, with mild (scores 0-10), moderate (scores 11-20), severe (scores 21-30), and most severe (scores 31-40). The total RLS score is analyzed as a continuous variable in this study, while the RLS index is treated as a categorical variable, classified based on severity using the aforementioned score ranges.

##### Dysfunctional Beliefs and Attitudes about Sleep

The DBAS is a self-reported questionnaire composed of 16 items with a Likert scale of 0-10. It assesses dysfunctional beliefs about and attitudes toward sleep, and the total score ranges from 0 to 160. The higher the total score, the more dysfunctional beliefs about and attitudes toward sleep one has.

##### State-Trait Anxiety Inventory–State

The STAI-S measures anxiety as a state with 20 items with a Likert scale of 1-4. The higher the score, the greater the tendency for anxiety one has.

##### Patient Health Questionnaire-9

The PHQ-9 is a widely used self-reported questionnaire designed to screen, diagnose, and assess the severity of depression. It is composed of 9 items with a Likert scale of 0-3. The total score ranges from 0 to 27, and scores are interpreted as the following: no depression (scores 0-4), mild depression (scores 5-9), moderate depression (scores 10-19), and severe depression (scores 20-27). The total PHQ-9 score is analyzed as a continuous variable in this study, while the PHQ-9 index is treated as a categorical variable, classified based on severity using the aforementioned score ranges.

##### Generalized Anxiety Disorder-7

The GAD-7 is a self-reported questionnaire screening for and measuring the severity of GAD symptoms. It includes 7 items with a Likert scale of 0-3. The total score ranges from 0 to 21, and scores are interpreted as follows: no anxiety (scores 0-4), mild anxiety (scores 5-9), moderate anxiety (scores 10-14), and severe anxiety (scores 15-21). The total GAD-7 score is analyzed as a continuous variable in this study, while the GAD-7 index is treated as a categorical variable, classified based on severity using the aforementioned score ranges.

##### Alcohol Use Disorders Identification Test-Consumption

The Alcohol Use Disorders Identification Test (AUDIT) is a useful screening tool for alcohol use disorders. It is composed of 10 items on alcohol consumption, alcohol dependence, and alcohol-related problems, with a Likert scale of 0-4, except for 2 items with 0, 2, and 4 points. The AUDIT-C is used in this study. It is a short version of AUDIT with a focus on the alcohol consumption domain. It is also used to screen people with hazardous drinking who may have an active alcohol use disorder. It is composed of the first 3 items of AUDIT with a Likert scale of 0-4. The total scale of AUDIT-C ranges from 0 to 12, and the cutoff values are 4 or more for men and 3 or more for women. The total AUDIT-C score is analyzed as a continuous variable in this study, with a higher score indicating a higher risk for hazardous drinking. AUDIT-C index is treated as a categorical variable, classified based on the cutoff values of 4 for men and 3 for women.

##### Smartphone Overuse Screening Questionnaire

The SOS-Q was developed as a screening questionnaire with an aim to distinguish individuals at high risk of smartphone overuse from casual users by Lee et al [27] in 2017. It comprises 28 items on a 4-point scale from 1 to 4 that identify smartphone use habits and screen for smartphone addiction risk. The cutoff score is 49, and scores higher than 49 indicate a high risk of smartphone addiction. In this study, scores below 49 indicate a low risk, and scores higher than 49 indicate a high risk.

##### Biological Rhythms Interview of Assessment in Neuropsychiatry

The BRIAN is a self-reported scale used to clinically evaluate disturbances in biological rhythm. It is composed of 21 items, and of these, 18 are included in the corresponding section of the following 4 categories related to circadian rhythm disturbances: sleep, social rhythms, activity, and eating patterns. The remaining 3 items collect information that addresses chronotype. All items are assessed with the Likert scale of 1-4, reflecting the frequency of problems related to the maintenance of a regular rhythm. The total BRIAN score ranges from 18 to 72, and the higher scores indicate more severe circadian rhythm disturbance.

##### Ecological Momentary Assessment Using the SOMDAY

The daily recordings of habits and lifestyle choices by the study participants using SOMDAY provided ecological momentary assessments. The SOMDAY, a smartphone app developed by our team, is compatible with both the Android OS and iOS platforms. It prompts its users to record their daily entries every day at 9 PM, and it collects information on alcohol consumption, caffeine intake, naps, stress level, self-reported sleep duration, and frequency of waking up during the night.

Alcohol consumption, caffeine intake, naps, and stress level were recorded in amount and timing. These 4 daily recordings were calculated using weights based on when they occurred. For caffeine intake, the occurrences in the morning, afternoon, and before bedtime were weighted by 1, 1.5, and 2, respectively. The variable caffeine intake, therefore, was obtained by applying the aforementioned weights based on the time of occurrence to the intake amount. In the case of naps, since both nap duration and nap occurrence are categorical data, we encoded nap duration as 1 for naps more than an hour and less than 2 hours, 2 for naps more than 2 hours and less than 3 hours, 3 for naps more than 3 hours, and 0.5 for naps less than an hour. Like the caffeine intake, weights were applied to the naps based on when they occurred: 1, 1.5, and 2 for morning, afternoon, and before bedtime, respectively. Stress level was encoded in intensity (mild, 1; moderate, 2; and severe, 3) and timing weighed such as the caffeine intake (morning, 1; afternoon, 1.5; and before bedtime, 2). The weights for the alcohol consumption timing were 1.5 for the morning, 1 for the afternoon, and 2 for before bedtime, because drinking in the morning was considered more harmful than drinking in the afternoon. The variable alcohol consumption therefore was obtained by applying the aforementioned weights based on the time of drinking to the alcohol consumption amount.

#### Objective Data: Digital Phenotypes From the Wearable Devices

Complementary to the subjective data collected from the self-reported questionnaires and ecological momentary assessment, the Fitbit devices worn by study participants automatically collected recording data about sleep time, heart rate, steps, and walking distance. The data were collected from March 2023 to November 2023, and 4-week data were collected for each participant. In addition, the heart rate data were recorded every 5 minutes, and sleep parameters were generated on a daily basis. Steps and walking distance were cumulative data, so we used differencing and day averages to get features and circadian rhythms.

Sleep parameters consisted of total sleep time, total awake time, number of wake-ups during the night, REM sleep time, light sleep time, and deep sleep time. We used the total sleep time and total awake time to calculate the sleep quality. Fitbit estimates sleep stages based on a combination of movement and heart rate patterns. Inactivity for about an hour is assumed to be sleep, and movements large enough to disturb sleep indicate wakefulness. Heart rate variability is used to further estimate the sleep stages, such as REM, light, and deep sleep. Total sleep time is defined as the total inactive time subtracted by the total awake time. The Fitbit-obtained sleep parameters are measured in minutes, except for the sleep quality which is calculated to be a percentage. For walking distance, we calculated the maximum, minimum, and average values per day.

Cosinor analysis was performed to analyze and extract circadian rhythms of heart rate [29]. We generated the main parameters using a 2-day time window: midline estimating static of rhythm (MESOR), amplitude, acrophase, and goodness of fit. MESOR represents the rhythm-adjusted mean, reflecting the average value of a parameter over the analyzed period. Amplitude is defined as the difference between the MESOR and the peak value, providing a quantitative measure of the extent of predictable variation within the circadian cycle. Acrophase refers to the timing of the peak value within each cycle, offering an insight into the degree of circadian misalignment. Finally, the goodness of fit is an indicator of how well the data fit to a 24-hour cosine model, reflecting the strength and clarity of the observed circadian rhythm. In addition, the maximum, minimum, and average heart rates per day were calculated.

Steps were analyzed in the same way as the heart rate, and the maximum, minimum, and average were calculated for weekdays and weekends and day and night, separately. To extract additional activity cycle information, least active 5-hour period (L5) and most active 10-hour period (M10), intradaily variability, and interdaily stability were calculated [30]. L5 and M10 were calculated using the moving average method. L5 is the minimum value with a time window of 5 hours, and M10 is the maximum value with a time window of 10 hours.

Cosinor analysis features and sleep data were calculated as average values over a 4-week period, and the maximum and minimum daily values were averaged by weekday and weekend and by day (8 AM to 6 PM) and night (6 PM to 8 AM). L5, M10, interdaily stability, and intradaily variability were averaged depending on weekdays and weekends.

### Statistical Analysis

First, a normality test of the 4 groups classified by the ISI score (no insomnia group, mild insomnia group, moderate insomnia group, and severe insomnia group) was performed. A Kruskal-Wallis test and a paired Wilcoxon’s rank sum test were performed when normal distribution could not be assumed. By the Bonferroni method, *P*<.0083 was considered statistically significant. When the normal distribution could be assumed, a one-way analysis of variance was performed for continuous data, and the Tukey honest significant different test was used for post hoc analysis. A chi-square test was used for the categorical data.

### Ethical Considerations

All study procedures were approved by the Institutional Review Board of Korea University Anam Hospital (2022AN0587), Seoul, Korea. Written informed consent was obtained from all the study participants at the beginning of the study. A compensation of 100,000 KRW (US $68.36) was provided for the completion of the 4-week study. The collected data were anonymized.

## Results

Based on the ISI score, the study participants were classified into the following 4 groups: the no insomnia group (scores 0-7), the mild insomnia group (scores 8-14), the moderate insomnia group (scores 15-21), and the severe insomnia group (scores 22-28). Of the total of 250 study participants, 63 (25.2%) were in the no insomnia group, 106 (42.4%) in the mild insomnia group, 69 (27.6%) in the moderate insomnia group, and 12 (4.8%) in the severe insomnia group. Although there were no significant differences across the 4 groups in the proportion of female sex (*P*=.79), the participants of the no insomnia group were significantly younger, with a median value of 25 (IQR 22-29) years, than those of the insomnia groups of all severity (mild: median 28, IQR 24-36 years; moderate: median 28, IQR 24-36 years; and severe: median 29, IQR 24.5-38 years; *P*=.02), as shown in Table 1.

**Table 1:** Demographic information and wearable device–obtained objective sleep parameters of the study participants.

Group		No insomnia (n=63)	Mild insomnia (n=106)	Moderate insomnia (n=69)	Severe insomnia (n=12)	*P* value	Post hoc analysis
**Demographic information**							
	Sex (female), n (%)^a^	40 (63.5)	69 (65.1)	49 (71.0)	8 (66.7)	.79	—^b^
	Age (years), median (IQR)^c^	25 (22-29)	28 (24-36)	28 (24-36)	29 (24.5-38)	.02	No insomnia group<insomnia groups
	BMI (kg/m^2^), median (IQR)^c^	21.77 (20.2-24.34)	21.84 (20.45-24.31)	21.79 (19.13-25.38)	23.49 (20.11-25.58)	.63	—
**Wearable device-obtained objective sleep parameters**							
	Total sleep time (min), median (IQR)^c^	375.48 (349.79-406.83)	378.96 (353.79-404.07)	372.01 (350.1-394.76)	384.97 (346.7-398.27)	.80	—
	Total awake time (min), median (IQR)^c^	69.52 (64-77.8)	63.84 (55.86-72.24)	63.22 (54.25-72.16)	58.76 (51.4-78.61)	.01	Mild and moderate insomnia groups<no insomnia group
	REM^d^ sleep time (min), median (IQR)^c^	210.04 (191.04-230.79)	209.36 (189.34-234.91)	212.43 (186.4-226.46)	199.2 (183.18-223.34)	.92	—
	Light sleep time (min), median (IQR)^c^	77.11 (64.9-91)	79.37 (69-91.17)	76.27 (61.39-86)	79.92 (62.41-93.58)	.32	—
	Deep sleep time (min), median (IQR)^c^	53.61 (48.52-60.14)	54.36 (46.57-60.16)	54.48 (47.46-60.96)	54.54 (47.01-62.84)	.93	—
	Number of wake-ups (per week), median (IQR)^c^	26.22 (23.97-29.71)	25.81 (22.23-29.54)	25 (20.77-28.42)	24.96 (20.41-28.56)	.51	—
	Sleep quality (%), median (IQR)^c^	82 (80-83)	83 (81-85)	83 (81-85)	83 (81-85)	.01	No insomnia group<insomnia groups
**Self-reported sleep parameter**							
	Naptime (min), median (IQR)^c^	3.75 (0-8.75)	3.5 (0-7.75)	5 (0.75-13.5)	1.5 (0-8.63)	.25	—

^a^Chi-square.

^b^Not applicable.

^c^Kruskal-Wallis test (multiple comparisons with Wilcoxon rank sum test with *P*<.0083 for 4 comparison groups).

^d^REM: rapid eye movement.

As for the sleep parameters obtained from the wearable devices (Table 1), there were no significant differences across the 4 groups in total sleep time, total awake time, REM sleep time, light sleep time, deep sleep time, number of wake-ups per week, and nap time (all *P*>.05). Interestingly, the participants of the no insomnia group had significantly longer total awake time with the median value of 69.52 (IQR 64-77.8) minutes than those of the mild and moderate insomnia groups (mild: median 63.84, IQR 55.86-72.24 minutes and moderate: median 63.22, IQR 54.25-72.16 minutes; *P*=.01). The participants of the no insomnia group also had a significantly lower sleep quality with the median value of 82% (IQR 80%-83%) than the insomnia groups (mild: median 83%, IQR 81%-85%; moderate: median 83%, IQR 81%-85%; and severe: median 83%, IQR 81%-85%; *P*=.01). In Table 2, although there were no significant differences in the circadian rhythm parameters between the no insomnia group and the insomnia groups (all *P*>.05), the amplitude values of both heart rate circadian rhythm and steps circadian rhythm were higher in the no insomnia group compared with the insomnia groups. Likewise, the goodness-of-fit values of both heart rate circadian rhythm and steps circadian rhythm were higher in the no insomnia group than the insomnia group.

**Table 2:** Circadian rhythm parameters across the no insomnia group and insomnia groups of 3 severities.

Group	No insomnia (n=63)	Mild insomnia (n=106)	Moderate insomnia (n=69)	Severe insomnia (n=12)	*P* value	Post hoc analysis
HR^a^ CR^b^ MESOR^c^, median (IQR)^c^	74.56 (70.5-80.07)	75.03 (69.54-79.93)	73.79 (70.27-79.39)	75.92 (69.44-77.69)	.98	—
HR CR amplitude, median (IQR)^d^	11.02 (9.02-13.28)	10.10 (8.12-11.73)	10.69 (9.05-12.59)	9.84 (7.47-11.46)	.30	—
HR CR acrophase, median (IQR)^d^	6.48 (3.65-9.35)	7.2 (4.22-10.29)	6.38 (3.14-9.89)	5.07 (2.75-6.73)	.39	—
HR CR goodness of fit, mean (SD)^e^	0.39 (0.12)	0.38 (0.12)	0.38 (0.1)	0.35 (0.11)	.74	—
Steps CR MESOR, median (IQR)^d^	142.14 (–6.4 to 245.56)	158.58 (65.01-286.2)	163.33 (84.15-261.93)	173.02 (96.38-259.51)	.65	—
Steps CR amplitude, median (IQR)^d^	373.57 (120.56-4055.27)	188.83 (107.72-695.18)	203.00 (113.18-652.73)	182.10 (154.59-1439.82)	.18	—
Steps CR acrophase, median (IQR)^d^	–11.24 (–36.49 to 4.42)	–7.97 (–22.12 to 2.08)	–7.28 (–21.51 to 4.71)	–8.35 (–19.56 to 1.67)	.78	—
Steps CR goodness of fit, median (IQR)^d^	0.19 (0.12-0.29)	0.16 (0.11-0.23)	0.18 (0.12-0.24)	0.17 (0.12-0.23)	.37	—
IV^f^, median (IQR)^d^	0.14 (0.03-0.55)	0.12 (0.02-0.44)	0.12 (0.03-0.47)	0.16 (0.04-0.46)	.92	—
IS^g^, median (IQR)^d^	0.42 (0.18-0.62)	0.47 (0.23-0.82)	0.51 (0.28-0.79)	0.65 (0.4-1.12)	.16	—
L5^h^, median (IQR)^a^	251.43 (144.57-331.94)	186.12 (106.55-354.03)	171.46 (107.79-295.76)	187.19 (103.85-301.83)	.25	—
M10^i^, median (IQR)^a^	732.29 (521.95-919.35)	696.62 (504.61-823.81)	692.46 (496.87-833.5)	640.8 (497.54-836.32)	.60	—

^a^HR: heart rate.

^b^CR: circadian rhythm.

^c^MESOR: midline estimating static of rhythm.

^d^Kruskal-Wallis test (multiple comparison with Wilcoxon rank sum test with *P*<.0083 for 4 comparison groups.

^e^ANOVA test (post hoc analysis performed with the Tukey honest significant different test).

^f^IV: intradaily variability.

^g^IS: interdaily stability.

^h^L5: least active 5-hour period.

^i^M10: most active 10-hour period.

In Table 3, the insomnia groups had significantly higher scores of the IRLS, DBAS, SOS-Q, BRIAN, STAI-S, PHQ-9, and GAD-7 than the no insomnia group. Within the 3 insomnia groups, the moderate and severe groups had a higher IRLS score than the mild group (*P*<.001), and the severe group had a higher stress score (*P*≤.001) and DBAS score (*P*<.001). Furthermore, the insomnia groups of different severities had significantly different scores in 5 of the above 7 questionnaires (SOS-Q, BRIAN, STAI-S, PHQ-9, and GAD-7), with the increasingly higher scores corresponding to the more severe subjective insomnia complaints. The SOS-Q scores were found to be increasingly higher corresponding to the severity of subjective insomnia, ranging from 44 (IQR 36-55) for the no insomnia group, 45 (IQR 38-60) for the mild insomnia group, 54 (IQR 42-64) for the moderate insomnia group, to 65 (IQR 50.5-72.5) for the severe insomnia group (*P*=.001). The scores of BRIAN, STAI-S, PHQ-9, and GAD-7 also showed the same pattern as the SOS-Q scores with a statistical significance (*P*<.001).

**Table 3:** Clinical scales on sleep-related health behaviors, cognition, neurological symptoms, and mood state.

Group			No insomnia (n=63)		Mild insomnia (n=106)	Moderate insomnia (n=69)		Severe insomnia (n=12)		*P* value			Post hoc analysis	
Caffeine intake, median (IQR)^a^			10 (1-21)		15.5 (0-30)	13 (1.5-25)		21 (4.25-40.25)		.42			—^b^	
Alcohol consumption, median (IQR)^a^			2 (0-18)		11 (0, 34)	4 (0, 23)		3.5 (0, 26.5)		.07			—	
AUDIT-C^c^ score, median (IQR)^a^			4 (2-7)		5 (2-8)	3 (1-7)		3.5 (0.5-7.50)		.54			—	
**AUDIT-C index^d^, n (%)**											.84			—
	Nonhazardous	21 (33.3)		38 (35.8)		28 (40.6)	5 (41.7)		—			—		
	Hazardous	42 (66.7)		68 (64.2)		41 (59.4)	7 (58.3)		—			—		
**Stress, median (IQR)^a^**			0 (0-13.5)		12 (0-43)	9 (1.5-36.5)		48.5 (1.75-152.75)		<.001			No insomnia<mild-to-moderate insomnia<severe insomnia	
**RLS^e^ score, median (IQR)^a^**			0 (0)		0 (0-14)	10 (0-18)		13.5 (0-24)		<.001			No insomnia<mild insomnia<moderate-to-severe insomnia	
**RLS index^d^, n (%)**											<.001			—
	Mild	61 (96.8)		73 (68.9)		35 (50.7)	6 (50.0)		—			—		
	Moderate	1 (1.6)		24 (22.6)		21 (30.4)	1 (8.3)		—			—		
	Severe	0 (0.0)		7 (6.6)		12 (17.4)	4 (33.3)		—			—		
	Most severe	1 (1.6)		2 (1.9)		1 (1.4)	1 (8.3)		—			—		
**DBAS^f^ score, mean (SD)^g^**			65.17 (23.56)		81.23 (20.07)	94.12 (19.36)		119.5 (26.68)		<.001			No insomnia<mild-to-moderate insomnia<severe insomnia	
**SOS-Q^h^ score, median (IQR)^a^**			44 (36-55)		45 (38-60)	54 (42-64)		65 (50.5-72.5)		.001			No insomnia<mild<moderate<severe insomnia	
**SOS-Q index^d^, n (%)**											<.001			—
	Low risk	44 (69.8)		61 (57.5)		27 (39.1)	3 (25.0)		—			—		
	High risk	19 (30.2)		45 (42.5)		42 (60.9)	9 (75.0)		—			—		
**BRIAN^i^ score, median (IQR)^a^**			34 (30-38)		43 (37-47)	49 (43-54)		52 (45.5-59)		<.001			No insomnia<mild<moderate<severe insomnia	
**STAI-S^j^ score, median (IQR)^a^**			32 (24-40)		43 (35-49)	47 (40-51)		58 (39-61.5)		<.001			No insomnia<mild<moderate<severe insomnia	
**PHQ-9^k^ score, median (IQR)^a^**			2 (1-4)		5 (3-8)	8 (5-12)		13.5 (8.5-17)		<.001			No insomnia<mild<moderate<severe insomnia	
**PHQ-9 index^d^, n (%)**											<.001			—
	Normal	53 (84.1)		47 (44.3)		14 (20.3)	2 (16.7)		—			—		
	Mild	9 (14.3)		42 (39.6)		30 (43.5)	2 (16.7)		—			—		
	Moderate	1 (1.6)		17 (16.0)		22 (31.9)	7 (58.3)		—			—		
	Severe	0 (0.0)		0 (0.0)		3 (4.3)	1 (8.3)		—			—		
**GAD-7^l^ score, median (IQR)^a^**			0 (0-2)		2 (1-5)	3 (2-6)		11.5 (3.5-14)		<.001			No insomnia<mild<moderate<severe insomnia	
**GAD-7 index^d^, n (%)**											<.001			—
	Normal	59 (93.7)		78 (73.6)		42 (60.9)	4 (33.3)		—			—		
	Mild	4 (6.3)		20 (18.9)		17 (24.6)	1 (8.3)		—			—		
	Moderate	0 (0.0)		6 (5.7)		10 (14.5)	5 (41.7)		—			—		
	Severe	0 (0.0)		2 (1.9)		0 (0.0)	2 (16.7)		—			—		

^a^Kruskal-Wallis test (multiple comparisons with Wilcoxon rank sum test with *P*<.0083 for 4 comparison groups).

^b^Not applicable.

^c^AUDIT-C: Alcohol Use Disorders Identification Test-Consumption.

^d^Chi-square test.

^e^RLS: restless legs syndrome.

^f^DBAS: Dysfunctional Beliefs and Attitudes About Sleep.

^g^ANOVA test (post hoc analysis performed with Tukey Honest Significant Different test).

^h^SOS-Q: Smartphone Overuse Screening Questionnaire.

^i^BRIAN: Biological Rhythms Interview of Assessment in Neuropsychiatry.

^j^STAI-S: State-Trait Anxiety Inventory–State.

^k^PHQ-9: Patient Health Questionnaire-9.

^l^GAD-7: Generalized Anxiety Disorder-7.

## Discussion

### Principal Findings

This study aimed to examine how the severity of insomnia, as measured by the ISI, reflects objective sleep quality and correlates with other sleep-related subjective and objective parameters, including mood state, substance use, circadian rhythm, sleep-related beliefs, and neurological symptoms. A distinctive feature and strength of this study is the use of digital phenotyping, leveraging wearable devices and smartphone apps to collect real-world, continuous data on participants’ sleep patterns and related behaviors. This digital phenotyping approach allows for a more comprehensive understanding of insomnia, capturing both physiological and behavioral aspects in a naturalistic environment.

The results indicated that there were no significant differences in general sleep structure, such as total sleep time and sleep stages, as measured by wearable devices, between individuals with no insomnia and those with varying levels of insomnia severity. This lack of significant differences might be due to the objective sleep parameters being collected using wearable devices and that subjective insomnia severity does not necessarily align with objectively measured sleep quality, emphasizing the importance of factors beyond traditional sleep metrics [31].

First, the objective sleep parameters in this study, including total sleep time, sleep stages, and circadian rhythm metrics, were measured using wearable devices rather than polysomnography, which may influence the interpretation of discrepancies observed between subjective and objective sleep assessments [11]. Digital phenotyping, through the use of wearable devices, provided continuous, real-world data that facilitated the assessment of sleep parameters without the constraints of sleep laboratories, which might not reflect natural sleep patterns [32]. This real-world approach may better capture the complexities of insomnia, which are often influenced by environmental and behavioral factors. The ability of digital phenotyping to provide both objective and subjective data highlights its potential for improving our understanding of insomnia’s multifaceted nature [33].

Second, a key finding was that participants in the insomnia groups had higher scores in measures of stress, dysfunctional beliefs about sleep, and symptoms of RLS compared with the no insomnia group. This highlights the potential role of psychological and physiological factors in shaping perceptions of insomnia severity. Stress and cognitive factors, such as dysfunctional beliefs about sleep, have been linked to increased sleep difficulties, which can exacerbate subjective complaints even in the absence of objective sleep disruptions [34,35]. Therefore, this finding underscores the necessity for treatment strategies that address these psychological elements, such as cognitive behavioral therapy for insomnia [36,37].

Furthermore, the participants in the insomnia groups had higher scores in measures of smartphone overuse, biological rhythm disturbance, anxiety as a state, depression, and generalized anxiety than the no insomnia group. Interestingly, the scores of such measures were significantly different among the insomnia groups as well; the higher the severity of subjective insomnia, the higher the scores of the measures. Another notable finding of this study is that such psychological and behavioral factors affect the perceived, subjective insomnia severity without making significant differences in the activity-based objective sleep parameters. Excessive smartphone use is consistently associated with poor sleep quality, depression, and anxiety [38-41]. Likewise, depression and anxiety are well-known risk factors for poor sleep quality. Depression, anxiety, and excessive smartphone use can lead to biological rhythm disturbances and vice versa. The intricate relationship among psychological distress, behaviors such as smartphone overuse, and biological rhythm disturbance remains to be revealed in future research.

Clinical implications of these findings include the importance of using both subjective and objective assessments in the diagnosis and treatment of insomnia. For example, cognitive behavioral therapy for insomnia, which has been shown to be effective in modifying dysfunctional sleep beliefs [42], may be particularly beneficial for patients whose subjective insomnia symptoms do not match objective findings. In addition, digital phenotyping could play a significant role in treatment monitoring and tailoring interventions based on individual patient needs. This capability is particularly advantageous when compared with traditional polysomnography, as wearable devices provide ongoing monitoring that can be used to assess changes in sleep patterns over time in a nonintrusive manner.

However, this study has several limitations. The reliance on wearable devices, while offering a practical method for assessing sleep in a naturalistic setting, may not provide the same level of accuracy as polysomnography, the gold standard in sleep assessment [43]. The American Academy of Sleep Medicine stated that “consumer sleep technologies cannot be used for the diagnosis and treatment of sleep disorders at this time” due to the lack of validation against polysomnography and US Food and Drug Administration clearance [44]. For example, sleep onset latency is found to be underestimated by Fitbit, leading to the possible overestimation of total sleep time. However, Fitbit overall showed high sensitivity (0.95-0.96) and relatively low specificity (0.58-0.69) for sleep detection in a meta-analysis of the accuracy of Fitbit and polysomnography, with no significant differences in wake time after sleep onset, total sleep time, and sleep efficiency [45]. Thus, Fitbit is considered a useful alternative to collecting objective sleep parameters and is widely used for research, including the All of Us research program and the Adolescent Brain Cognitive Development study of the National Institutes of Health [46,47]. Future studies that further integrate both polysomnographic and wearable device data to provide a more comprehensive view of sleep and to validate the findings from wearable technologies are warranted to overcome the current limitations of wearable devices in measuring sleep parameters. In addition, the sleep parameters collected across the no insomnia group and insomnia groups in this study were through the uniformly applied Fitbit and its algorithm. Even though the measurement reliability of sleep parameters obtained from Fitbit is yet to be sufficiently validated, there were no device-related deviations when comparing sleep parameters across the groups. Since evaluating differences across the groups was the main interest, rather than examining the values of sleep parameters, comparing the groups using uniformly measured parameters serves its purpose.

Furthermore, the study sample, predominantly composed of volunteers recruited from specific settings, may limit the generalizability of the findings to the broader population. The two-site recruitment may lead to a potential bias, and a younger population is prone to participate in the study due to their accessibility to and familiarity with the digital technology in this naturalistic study design. Given these characteristics and limitations of the study sample, caution is required when interpreting the results. As an important contributor to and possible consequence of insomnia, naps were collected as self-reported sleep behavior recorded by the study participants using the SOMDAY app. In this study, nap was measured as a categorical variable, and it would have been more informative if objective, continuous naptime data had been included.

Future research should clearly delineate the data sources, including wearable devices and polysomnography, to avoid potential misinterpretations of the sleep metrics used. In addition, future research should explore the relationship between psychological factors, such as anxiety and stress, and their impact on both subjective and objective sleep parameters using digital phenotyping methods. Studies that include diverse populations and combine multiple methods of sleep assessment will be crucial for understanding the full spectrum of insomnia. Moreover, further exploration of the role of circadian rhythms in subjective sleep complaints could help to develop chronotherapy-based interventions that may be effective for certain subsets of patients with insomnia. Digital phenotyping could also be used to assess circadian disruptions in real time and provide personalized interventions.

In summary, this study highlights the complex interplay between subjective insomnia severity and objective sleep parameters and underscores the strengths of using digital phenotyping to assess these relationships in real-world settings. The lack of significant differences in objective sleep across insomnia severities suggests that insomnia is influenced by factors beyond sleep quantity and quality, particularly psychological elements such as stress, dysfunctional beliefs, depression, and anxiety. These findings advocate for a comprehensive treatment approach that includes both cognitive and physiological aspects of sleep disturbances, facilitated by the capabilities of digital phenotyping.

### Conclusions

In conclusion, this study underscores the complex interplay between subjective insomnia severity and objective sleep parameters, highlighting the value of digital phenotyping in understanding these relationships in real-world settings. The findings reveal that subjective perceptions of insomnia are often not aligned with objective sleep metrics, emphasizing the role of psychological factors, such as stress, dysfunctional beliefs, depression, and anxiety, in shaping insomnia severity. This discrepancy points to the need for comprehensive treatment strategies that integrate both cognitive and physiological aspects of sleep disturbances.

Digital phenotyping, using wearable devices and smartphone apps, offers a unique advantage in capturing continuous, naturalistic sleep data, providing insights that are not possible through traditional sleep laboratory settings alone. Future research and clinical interventions should continue to leverage these technologies to develop personalized and effective treatment approaches for individuals with insomnia, addressing both their subjective experiences and objective sleep health.

## Appendix

Multimedia Appendix 1 Additional circadian rhythm parameters across the no insomnia group and insomnia groups of three severities.

## Abbreviations

AUDIT: Alcohol Use Disorders Identification Test
AUDIT-C: Alcohol Use Disorders Identification Test-Consumption
BRIAN: Biological Rhythms Interview of Assessment In Neuropsychiatry
DBAS: Dysfunctional Beliefs and Attitudes About Sleep
GAD-7: Generalized Anxiety Disorder-7
IRLS: International Restless Legs Scale
ISI: Insomnia Severity Index
L5: least active 5-hour period
M10: most active 10-hour period
MESOR: midline estimating static of rhythm
PHQ-9: Patient Health Questionnaire-9
RLS: restless legs syndrome
STAT-S: State-Trait Anxiety Inventory–State
SOS-Q: Smartphone Overuse Screening Questionnaire
STAI-S: State-Trait Anxiety Inventory-State

## Data Availability

The datasets generated during and/or analyzed during this study are available from the corresponding author on reasonable request.

## References

[1] (2013). Diagnostic and Statistical Manual of Mental Disorders, Fifth Edition.

[2] Sateia MJ (2014). International classification of sleep disorders-third edition: highlights and modifications. Chest.

[3] Simon GE, VonKorff M (1997). Prevalence, burden, and treatment of insomnia in primary care. Am J Psychiatry.

[4] Mai E, Buysse DJ (2008). Insomnia: prevalence, impact, pathogenesis, differential diagnosis, and evaluation. Sleep Med Clin.

[5] Roth T (2007). Insomnia: definition, prevalence, etiology, and consequences. J Clin Sleep Med.

[6] Ahn E, Baek Y, Park JE, Lee S, Jin HJ (2024). Elevated prevalence and treatment of sleep disorders from 2011 to 2020: a nationwide population-based retrospective cohort study in Korea. BMJ Open.

[7] Morin CM, Belleville G, Bélanger L, Ivers H (2011). The Insomnia Severity Index: psychometric indicators to detect insomnia cases and evaluate treatment response. Sleep.

[8] Savard MH, Savard J, Simard S, Ivers H (2005). Empirical validation of the Insomnia Severity Index in cancer patients. Psychooncology.

[9] Kawai K, Iwamoto K, Miyata S, Okada I, Ando M, Fujishiro H, Noda A, Ozaki N (2022). A study of factors causing sleep state misperception in patients with depression. Nat Sci Sleep.

[10] Castelnovo A, Ferri R, Punjabi NM, Castronovo V, Garbazza C, Zucconi M, Ferini-Strambi L, Manconi M (2019). The paradox of paradoxical insomnia: a theoretical review towards a unifying evidence-based definition. Sleep Med Rev.

[11] Guillodo E, Lemey C, Simonnet M, Walter M, Baca-García E, Masetti V, Moga S, Larsen M, Ropars J, Berrouiguet S, HUGOPSY Network (2020). Clinical applications of mobile health wearable-based sleep monitoring: systematic review. JMIR Mhealth Uhealth.

[12] Rykov Y, Thach TQ, Bojic I, Christopoulos G, Car J (2021). Digital biomarkers for depression screening with wearable devices: cross-sectional study with machine learning modeling. JMIR Mhealth Uhealth.

[13] Cho CH (2023). Revolutionizing sleep health: the promise and challenges of digital phenotyping. Chronobiol Med.

[14] Kim WP, Kim HJ, Pack SP, Lim JH, Cho CH, Lee HJ (2023). Machine learning-based prediction of attention-deficit/hyperactivity disorder and sleep problems with wearable data in children. JAMA Netw Open.

[15] Cho CH, Lee T, Kim MG, In HP, Kim L, Lee HJ (2019). Mood prediction of patients with mood disorders by machine learning using passive digital phenotypes based on the circadian rhythm: prospective observational cohort study. J Med Internet Res.

[16] Lee HJ, Cho CH, Lee T, Jeong J, Yeom JW, Kim S, Jeon S, Seo JY, Moon E, Baek JH, Park DY, Kim SJ, Ha TH, Cha B, Kang HJ, Ahn YM, Lee Y, Lee JB, Kim L (2022). Prediction of impending mood episode recurrence using real-time digital phenotypes in major depression and bipolar disorders in South Korea: a prospective nationwide cohort study. Psychol Med.

[17] Feehan LM, Geldman J, Sayre EC, Park C, Ezzat AM, Yoo JY, Hamilton CB, Li LC (2018). Accuracy of Fitbit devices: systematic review and narrative syntheses of quantitative data. JMIR Mhealth Uhealth.

[18] Kang SG, Kang JM, Ko KP, Park SC, Mariani S, Weng J (2017). Validity of a commercial wearable sleep tracker in adult insomnia disorder patients and good sleepers. J Psychosom Res.

[19] Haghayegh S, Khoshnevis S, Smolensky MH, Diller KR, Castriotta RJ (2019). Accuracy of wristband Fitbit models in assessing sleep: systematic review and meta-analysis. J Med Internet Res.

[20] Lim SE, Kim HS, Lee SW, Bae KH, Baek YH (2023). Validation of Fitbit inspire 2 against polysomnography in adults considering adaptation for use. Nat Sci Sleep.

[21] Walters AS, LeBrocq C, Dhar A, Hening W, Rosen R, Allen RP, Trenkwalder C (2003). Validation of the International Restless Legs Syndrome Study Group rating scale for restless legs syndrome. Sleep Med.

[22] Morin CM, Vallières A, Ivers H (2007). Dysfunctional Beliefs and Attitudes About Sleep (DBAS): validation of a brief version (DBAS-16). Sleep.

[23] Hahn D (1996). Korean adaptation of Spielberger's STAI (K-STAI). Korean Journal of Health Psychology.

[24] Park SJ, Choi HR, Choi JH, Kim K, Hong J (2010). Reliability and validity of the Korean version of the Patient Health Questionnaire-9 (PHQ-9). Anxiety and mood.

[25] Lee SH, Shin C, Kim H, Jeon SW, Yoon HK, Ko YH, Pae CU, Han C (2022). Validation of the Korean version of the Generalized Anxiety Disorder 7 self-rating scale. Asia Pac Psychiatry.

[26] Chang JW, Kim JS, Jung JG, Kim SS, Yoon SJ, Jang HS (2016). Validity of Alcohol Use Disorder Identification Test-Korean revised version for screening alcohol use disorder according to Diagnostic and Statistical Manual of Mental Disorders, Fifth Edition criteria. Korean J Fam Med.

[27] Lee HK, Kim JH, Fava M, Mischoulon D, Park JH, Shim EJ, Lee EH, Lee JH, Jeon HJ (2017). Development and validation study of the Smartphone Overuse Screening Questionnaire. Psychiatry Res.

[28] Cho CH, Jung SY, Kapczinski F, Rosa AR, Lee HJ (2018). Validation of the Korean version of the Biological Rhythms Interview of Assessment in Neuropsychiatry. Psychiatry Investig.

[29] Cornelissen G (2014). Cosinor-based rhythmometry. Theor Biol Med Model.

[30] Krafty RT, Fu H, Graves JL, Bruce SA, Hall MH, Smagula SF (2019). Measuring variability in rest-activity rhythms from actigraphy with application to characterizing symptoms of depression. Stat Biosci.

[31] Bianchi MT, Williams KL, McKinney S, Ellenbogen JM (2013). The subjective-objective mismatch in sleep perception among those with insomnia and sleep apnea. J Sleep Res.

[32] Tobin SY, Williams PG, Baron KG, Halliday TM, Depner CM (2021). Challenges and opportunities for applying wearable technology to sleep. Sleep Med Clin.

[33] Perez-Pozuelo I, Zhai B, Palotti J, Mall R, Aupetit M, Garcia-Gomez JM, Taheri S, Guan Y, Fernandez-Luque L (2020). The future of sleep health: a data-driven revolution in sleep science and medicine. NPJ Digit Med.

[34] Harvey AG (2002). A cognitive model of insomnia. Behav Res Ther.

[35] Harvey CJ, Gehrman P, Espie CA (2014). Who is predisposed to insomnia: a review of familial aggregation, stress-reactivity, personality and coping style. Sleep Med Rev.

[36] Alimoradi Z, Jafari E, Broström A, Ohayon MM, Lin CY, Griffiths MD, Blom K, Jernelöv S, Kaldo V, Pakpour AH (2022). Effects of cognitive behavioral therapy for insomnia (CBT-I) on quality of life: a systematic review and meta-analysis. Sleep Med Rev.

[37] Edinger JD, Arnedt JT, Bertisch SM, Carney CE, Harrington JJ, Lichstein KL, Sateia MJ, Troxel WM, Zhou ES, Kazmi U, Heald JL, Martin JL (2021). Behavioral and psychological treatments for chronic insomnia disorder in adults: an American Academy of Sleep Medicine clinical practice guideline. J Clin Sleep Med.

[38] Demirci K, Akgönül M, Akpinar A (2015). Relationship of smartphone use severity with sleep quality, depression, and anxiety in university students. J Behav Addict.

[39] Thomée S, Härenstam A, Hagberg M (2011). Mobile phone use and stress, sleep disturbances, and symptoms of depression among young adults--a prospective cohort study. BMC Public Health.

[40] Yang J, Fu X, Liao X, Li Y (2020). Association of problematic smartphone use with poor sleep quality, depression, and anxiety: a systematic review and meta-analysis. Psychiatry Res.

[41] Chung JE, Choi SA, Kim KT, Yee J, Kim JH, Seong JW, Seong JM, Kim JY, Lee KE, Gwak HS (2018). Smartphone addiction risk and daytime sleepiness in Korean adolescents. J Paediatr Child Health.

[42] Espie CA, Inglis SJ, Tessier S, Harvey L (2001). The clinical effectiveness of cognitive behaviour therapy for chronic insomnia: implementation and evaluation of a sleep clinic in general medical practice. Behav Res Ther.

[43] Mantua J, Gravel N, Spencer RMC (2016). Reliability of sleep measures from four personal health monitoring devices compared to research-based actigraphy and polysomnography. Sensors (Basel).

[44] Khosla S, Deak MC, Gault D, Goldstein CA, Hwang D, Kwon Y, O'Hearn D, Schutte-Rodin S, Yurcheshen M, Rosen IM, Kirsch DB, Chervin RD, Carden KA, Ramar K, Aurora RN, Kristo DA, Malhotra RK, Martin JL, Olson EJ, Rosen CL, Rowley JA (2018). Consumer sleep technology: an American Academy of Sleep Medicine position statement. J Clin Sleep Med.

[45] Haghayegh S, Khoshnevis S, Smolensky MH, Diller KR, Castriotta RJ (2020). Performance assessment of new-generation Fitbit technology in deriving sleep parameters and stages. Chronobiol Int.

[46] Munos B, Baker PC, Bot BM, Crouthamel M, de Vries G, Ferguson I, Hixson JD, Malek LA, Mastrototaro JJ, Misra V, Ozcan A, Sacks L, Wang P (2016). Mobile health: the power of wearables, sensors, and apps to transform clinical trials. Ann N Y Acad Sci.

[47] Lee HA, Lee HJ, Moon JH, Lee T, Kim MG, In H, Cho CH, Kim L (2017). Comparison of wearable activity tracker with actigraphy for sleep evaluation and circadian rest-activity rhythm measurement in healthy young adults. Psychiatry Investig.

