# Supplementary material for: Exploring the Psychological and Physiological Insights Through Digital Phenotyping by Analyzing the Discrepancies Between Subjective Insomnia Severity and Activity-Based Objective Sleep Measures: Observational Cohort Study
Source: JMIR Ment Health. 2025 Jan 27;12:e67478. doi: 10.2196/67478 (PMC11811666; doi:10.2196/67478)
Supplement: Multimedia Appendix 1 [file mental_v12i1e67478_app1.docx]

|  | No insomnia  (n=63) | Mild insomnia  (n=106) | Moderate insomnia  (n=69) | Severe insomnia  (n=12) | *P* value | Post hoc analysis |
| --- | --- | --- | --- | --- | --- | --- |
| Minimum day HR^a^, mean (SD)^c^ | 52.03 (6.07) | 52.75 (6.25) | 52.93 (5.57) | 53.75 (4.59) | 0.74 | __ |
| Maximum day HR^a^, mean (SD)^c^ | 148.2 (19.37) | 143.58 (19.31) | 145.74 (19.15) | 150.75 (22.21) | 0.38 | __ |
| Mean day HR^a^, mean (SD)^c^ | 80.23 (8.6) | 80.17 (8.17) | 79.18 (6.3) | 81.03 (5.44) | 0.78 | __ |
| Minimum night HR^a^, median (IQR)^b^ | 49 (46-51) | 49.5 (45-52) | 50 (47-53) | 50 (45.5-53.5) | 0.57 | __ |
| Maximum night HR^a^, median (IQR)^b^ | 145 (132-165) | 144.5 (128-158) | 143 (131-157) | 153 (129-168) | 0.68 | __ |
| Mean night HR^a^, median (IQR)^b^ | 71.5 (66.95-75.05) | 71.4 (65.49-75.79) | 71.54 (67.87-77.06) | 72.64 (66.79-74.27) | 0.96 | __ |
| Minimum day distance (m), median (IQR)^b^ | 10 (10-10) | 10 (10-10) | 10 (10-10) | 10 (10-10) | 0.10 | __ |
| Maximum day distance (m), median (IQR)^b^ | 2340 (1310-3350) | 2160 (1350-3740) | 2030 (1150-3470) | 2980 (1340-3830) | 0.52 | __ |
| Mean_day distance (m), median (IQR)^b^ | 190 (140-240) | 190 (140-240) | 180 (140-230) | 190 (160-290) | 0.74 | __ |
| Minimum night distance (m), median (IQR) ^b^ | 10 (10-10) | 10 (10-10) | 10 (10-10) | 10 (10-10) | 0.89 | __ |
| Maximum night distance (m), median (IQR)^b^ | 3410 (1690-6480) | 2930 (130-6150) | 250 (1420-5010) | 2980 (1280-6000) | 0.44 | __ |
| Mean night distance (m), median (IQR)^b^ | 190 (150-280) | 160 (100-260) | 160 (120-230) | 190 (130-220) | 0.14 | __ |

**Multimedia table 1. Additional circadian rhythm parameters across the no insomnia group and insomnia groups of three severities**

^a^HR: heart rate.

^b^Kruskal-Wallis test (multiple comparison with Wilcoxon rank sum test with P<.0083 for 4 comparison groups.

^c^ANOVA test (post hoc analysis performed with the Turkey honest significant different test).
